# Prevention of multiple system atrophy using human bone marrow-derived mesenchymal stem cells by reducing polyamine and cholesterol-induced neural damages

**DOI:** 10.1186/s13287-020-01590-1

**Published:** 2020-03-04

**Authors:** Kyung-Ran Park, Chul Ju Hwang, Hyung-Mun Yun, In Jun Yeo, Dong-Young Choi, Pil-Hoon Park, Hyung Sook Kim, Jung Tae Lee, Young Suk Jung, Sang-Bae Han, Jin Tae Hong

**Affiliations:** 1 Department of Oral and Maxillofacial Pathology, School of Dentistry, Kyung Hee University, Seoul, 02453 Republic of Korea; 2 College of Pharmacy and Medical Research Center, Chungbuk National University, 194-31, Osongsangmyeong1-ro, Heungdeok-gu, Cheongju, Chungbuk 361-951 Republic of Korea; 3 College of Pharmacy, Yeungnam University, 280, Daehak-ro, Gyeongsan, Gyeongbuk 712-749 Republic of Korea; 4 Corestem Inc, Pangyo-ro 255 beon-gil, Bundang-gu, Seongnam-si, Gyeonggi 13486 Republic of Korea; 5 College of Pharmacy, Pusan National University, Geumjeong-gu, Busan, Republic of Korea

**Keywords:** Cholesterol, MSC, Multiple system atrophy (MSA), Polyamines

## Abstract

**Background:**

Multiple system atrophy (MSA) is a sporadic neurodegenerative disorder of unknown etiology, but is closely associated with damage to dopaminergic neurons. MSA progression is rapid. Hence, long-term drug treatments do not have any therapeutic benefits. We assessed the inhibitory effect of mesenchymal stem cells (MSCs) on double-toxin-induced dopaminergic neurodegenerative MSA.

**Results:**

Behavioral disorder was significantly improved and neurodegeneration was prevented following MSC transplantation. Proteomics revealed lower expression of polyamine modulating factor-binding protein 1 (PMFBP1) and higher expression of 3-hydroxymethyl-3-methylglutaryl-CoA lyase (HMGCL), but these changes were reversed after MSC transplantation. In the in vitro study, the 6-OHDA-induced effects were reversed following co-culture with MSC. However, PMFBP1 knockdown inhibited the recovery effect due to the MSCs. Furthermore, HMGCL expression was decreased following co-culture with MSCs, but treatment with recombinant HMGCL protein inhibited the recovery effects due to MSCs.

**Conclusions:**

These data indicate that MSCs protected against neuronal loss in MSA by reducing polyamine- and cholesterol-induced neural damage.

## Background

Multiple system atrophy (MSA) is an adult-onset, sporadic, rapidly progressive, multisystem, neurodegenerative fatal disease of undetermined etiology. The neuropathological hallmark of MSA is cell loss in the striatonigral region of the brain [1]. Upon pathological examination, MSA exhibits striatonigral and olivopontocerebellar degeneration and bone marrow destruction [2]. In clinical settings, autonomic failure, parkinsonism, and cerebellar ataxia were observed in such cases [1]. The double-toxin-induced MSA model was first proposed in 1996. This model showed dopaminergic neurodegeneration following injections of 6-hydroxydopamine hydrochloride (6-OHDA) into the medial forebrain bundle, as well as ipsilateral excitotoxic striatal degeneration following injections of quinolinic acid (QA) [3]. The significant loss of dopaminergic neuronal cells, induced by the two toxins, leads to dopamine deficiency, which can consequently result in behavioral abnormalities.

Mesenchymal stem cells (MSCs) are multipotent stem cells that are capable of self-renewal and differentiation into a wide range of tissues [4]. MSCs exhibit higher proliferative capacity and safety [5, 6], and demonstrate non-invasive and ethically non-problematic availability [7]. Recently, several studies have described the applications of MSCs derived from adipose stem cells and bone marrow for neural disorders [8, 9]. Moreover, MSC grafts have demonstrated the potential for re-innervation of the striatum and amelioration of behavioral deficits in animal models of Parkinson’s disease (PD) [10].

Recently, the transplantation of MSCs into the brains of transgenic models of Alzheimer’s disease has been shown to alleviate symptoms [11–13]. MSCs also play various roles in the body and cellular environment, particularly in the maintenance of other cells [14]. Several in vitro and in vivo studies have shown that MSCs possess immunoregulatory properties. In vitro studies suggest that MSCs can inhibit the immune response and can release various soluble factors that might be involved in immunosuppressive activity [15–17]. Recent in vivo studies also demonstrated that MSC transplantation decreases the production of inflammatory cytokines and genes [18, 19]. These studies suggest that the anti-inflammatory and immunosuppressive action of MSCs is one possible mechanism underlying those protective effects. These properties of MSC may be useful in the treatment of several human neuronal diseases. Eleven MSA patients who underwent MSC transplantation intra-arterially for 3 months demonstrated a delay in the progression of neurological deficits [20]. Other human clinical trials have suggested that MSCs are also effective in neurodegenerative diseases such as PD [21] and amyotrophic lateral sclerosis [22, 23]. MSCs transplanted into the sublateral ventricular zone via stereotaxic surgery improved the disease status of PD patients [21]. The administration of MSCs into the hippocampus via stereotactic injection was feasible, safe, and well-tolerated in nine patients with Alzheimer’s disease (AD) [24]. At present, approximately 17 registered clinical trials are assessing the use of MSC therapy for MS treatment (http://www.clinicaltrials.gov/).

The increased levels of polyamines in the brain are associated with neurodegenerative diseases such as PD. Polyamines can induce DNA damage and can lead to cell death [25]. In fact, increased polyamine synthesis has been observed following inflammation [26]. A recent study showed that treatment with lipopolysaccharide (LPS) increased the synthesis of polyamines in cultured microglia [27]. A high level of polyamines consequently led to the accumulation of pro-inflammatory cytokines and chemokines such as TNF-α and CCL2. However, the inhibition of polyamine synthesis through the injection of a specific inhibitor prevented the induction of CCL2 expression by LPS [27]. Moreover, the bacterial infection has been demonstrated to increase the expression and activity of polyamine biosynthetic enzymes [28]. However, the LPS-induced neurodegeneration was reduced in mice with inhibited polyamine synthesis [29]. Polyamines are converted into putrescine by spermidine/spermine N1-acetyltransferase (SSAT1), which is expressed at a lower level in patients with PD. Polyamine modulating factor-binding protein 1 (PMFBP1) can enhance the catabolism and recycling of polyamines, thereby lowering levels of spermidine and spermine [30]. Thus, a strategy for lowering polyamine levels through the upregulation of PMFBP1 expression has received considerable interest in the amelioration of neurodegenerative diseases.

HMG-CoA lyase (HMGCL) is a mitochondrial matrix protein from the HMG-CoA lyase family of proteins [31]. Acetyl CoA acts as the precursor in the first stage of cholesterol synthesis. Recent studies have shown that cholesterol can exacerbate neurodegenerative pathology [32–36]. Moreover, studies on transgenic mouse models of AD indicated that high cholesterol levels can increase AD-pathology hallmarks such as amyloid levels, Tau phosphorylation, and behavioral deficits [33, 35, 37]. Some other prospective studies have found that high serum cholesterol levels may increase the risk of ischemic stroke [38]. Furthermore, recent studies have also shown that excess cholesterol accumulation induces the secretion of pro-inflammatory cytokines and stimulates macrophage recruitment [39]. In vitro studies have shown that cholesterol overload can induce endoplasmic reticulum stress and trigger the unfolded protein response, thus resulting in the activation of both c-Jun N-terminal kinases and IκB kinase, which are mediators of inflammatory cytokine production. High-cholesterol diets reportedly exacerbate various inflammatory diseases [40, 41]. In the brain, a high cholesterol diet increased oxidative stress in the hippocampus [42] and cortex [43], and also has been shown to elevate the expression of pro-inflammatory cytokines in the hippocampus of 9- to 11-month-old rats [44].

MSCs also regulate the expression of various genes or proteins during the development stage, so it can be used to treat many diseases. MSCs differentiate into adipocytes and express various genes, such as chitinase 3-like 1 (cartilage glycoprotein-39; CHI3L1), retinoic acid receptor responder (tazarotene induced 1; RARRES1), and immunoglobulin domain (Ig) [45]. Moreover, rats with MSC transplantation showed decreased expression of glial fibrillary acidic protein expression (GFAP) and oligodendroglial cell maturation [46]. These data indicate that MSCs may not only directly influence neuronal disease via self-renewal, but may also regulate gene or protein expression related to the inflammation and immune system. However, the direct role and mechanism, particularly the signaling mechanisms, of MSCs remain unclear.

In the present study, we aimed to assess whether the transplantation of human-derived MSCs could have beneficial effects in a double-toxin-induced MSA rat model. Moreover, we assessed the signaling-based mechanisms underlying the neuro-protective effects of MSCs.

### Materials

The present study conformed to the National Institute of Toxicological Research of the Korea Food and Drug Administration guidelines for the humane care and use of laboratory animals, and all the experimental animal procedures were performed strictly in accordance with the protocol approved by Ethical Committee, IACUC of Chungbuk National University (approval number CBNUA-144-1001-01).

### Animal experiments

A total of 60 adult male Wistar rats weighing 200–250 g were purchased from DBL (Seoul, Korea), and were maintained in accordance with the guidelines of the National Institute of Toxicological Research and Korea Food and Drug Administration for the humane care and use of laboratory animals. Animals were housed in a room that was automatically maintained at 21–25 °C, with a relative humidity of 45–65% and controlled 12-h light/dark cycle. All experimental procedures in the present study were approved by the IACUC of Chungbuk National University (approval number CBNUA-144-1001-01).

To induce MSA, the rats were randomly allocated to 6 groups (*n* = 10 each). Group 1 (control group) received saline, and group 2 (double-toxin group) received both QA and 6-OHDA. Groups 3 and 4 received MSC transplantation via intra-arterial (IA) injection (group 3, 1.2 × 10^5^; group 4, 6 × 10^5^), and groups 5 and 6 received MSC transplantation via intrathecal (IT) injection (group 5, 1.2 × 10^5^; group 6, 6 × 10^5^) after double-toxin induction.

### Lesion surgery

Rats were anesthetized with 40 mg/kg sodium pentobarbital isoflurane and placed in a stereotactic frame (Narishige, Japan). QA (Sigma) was dissolved at a concentration of 5 mg/mL in 1 M NaOH, followed by 0.1 M phosphate-buffered saline (PBS), and the pH was adjusted to 7.4. A total dose (5 μg/mL; equivalent to 90 nmol) was injected into each animal. Furthermore, 6-OHDA (Sigma) was dissolved at a concentration of 5 mg/mL in 0.9% NaCl with 0.2% ascorbic acid, and a total dose of 10 μg in 10 μL was injected in each animal. The unilateral stereotactic injection site included the right lateral striatum, which receives dense projections from the primary motor cortex and dopaminergic innervation exclusively from the nigra [47–49]. The coordinates were measured anterior and lateral to the bregma and vertical to the dura, with the tooth bar set at 3.3 mm below the interaural line. The coordinates of the lesion site were: anterior-5, 0 mm; lateral-5, 3.5 mm; and vertical-5, 5.5 mm, according to the Paxinos and Watson atlas. The toxins were infused at a rate of 5 μL/min through a 29-gauge stainless-steel cannula, attached via a polyethylene tubing to a glass syringe mounted in a Harvard microdrive pump. For each track, the cannula was initially placed stereotactically at the lower depth and left in place 5 min before the infusion. The cannula was maintained in place for an additional 5 min to allow for diffusion of the toxin or vehicle before it was slowly withdrawn and rinsed with distilled water prior to the second injection procedure. The wound was then cleaned and sutured, and the rat was allowed to recover. No additional postoperative care was required.

### Isolation and expansion of MSCs

MSCs derived from human patient bone marrow were obtained from Corestem Inc. (Seoul, Republic of Korea). In brief, bone marrow was aspirated from the posterior iliac crest of healthy donors, and mononuclear cells were collected by density gradient methods. The mononuclear cells were cultured in CSBM-A06 medium (Corestem Inc.) containing 10% fetal bovine serum (Gibco, Grand Island, NY, USA), 2.5 mM L-glutamine, and penicillin/streptomycin (WelGene, Gyeongsangbuk-do, Republic of Korea) in an incubator at 37 °C and 5% CO2 for 3–5 passages. After washing out non-adherent cells, the adherent cells retained the canonical phenotype of MSCs (CD29^+^CD44^+^CD73^+^CD105^+^CD90^+^CD34^−^CD45^−^HLA^−^DR^−^) and were used in the experiments. The study was approved by the Institutional Review Board of Hanyang University Hospital.

### Rotarod test

Motor performance and coordination were examined using the Rotarod treadmill (MED Associates Inc., St. Albans, VT, USA), which consisted of a 5-cm-diameter cylindrical treadmill connected to a computer-controlled stepper motor, as described previously [50]. The time at which the animal falls off the rotating drum was detected by individual sensors, and the time spent by the animal on the treadmill (in seconds) was automatically recorded. Rats were trained for 2 consecutive days before the double-toxin injections were given, in acceleration mode (5–30 rpm) over 5 min. The training was repeated at a fixed speed (15 rpm) until the rats were able to stay on the rod for at least 300 s. If the animals did not pass the training, they were excluded from further experiments.

### Gait test

Stride length was measured according to the method of Fernagut et al. [51]. In brief, after their forelimbs and hindlimbs were painted with ink, the animals were placed on a bright runway (9 cm wide and 90 cm long, with 20 cm high walls) and were allowed to run towards a dark goal box (40 × 30 × 20 cm). Rats were subjected to two training trials for acclimatization to the environment. A single test trial was performed and stride length was measured as the distance between successive paw prints. Data are presented as the average value of five strides for each animal.

### Grip strength

The maximal muscle strength of the forelimbs was measured by using an isometric transducer attached to a 3-mm-diameter metal bar (Ugo Basile). For the measurement of forelimb grip strength, each mouse gripped the bar with its forelimbs and was then slowly pulled backward until it released the metal bar. The transducer measured the maximal grip strength in grams. Five trials were performed in each testing session, and the maximum value was calculated.

### Brain tissue collection and preservation

After behavioral tests were conducted, the animals were perfused with PBS after being anesthetized by inhaled sodium pentobarbital isoflurane. The brains were immediately removed from the skull, and the cortex and hippocampus were dissected on ice. All the brain tissues were immediately stored at − 80 °C until biochemical assays could be conducted.

### Neuronal cell and microglial BV-2 cell culture

The Sprague-Dawley rats were maintained in accordance with the policy of the National Institute of Toxicological research, which was in accordance with the Korea Food and Drug Administration’s guideline for the care and use of laboratory animals. Sprague-Dawley rats weighing 200–300 g were housed under 12 h light/dark cycles at 23 °C and 60 ± 5% humidity. All animals had free access to food (Samyang Foods, Seoul, Republic of Korea) and water. Cerebral cortical cells were isolated from neonatal rat brains (day 1) in PBS (0.1 mol). Briefly, cerebral cortices were removed and incubated for 15 min in Ca^2+^- and Mg^2+^-free Hanks’ balanced saline solution (Life Technologies) containing 0.2% trypsin. Cells were dissociated by trituration and plated into polyethyleneimine-coated plastic or glass-bottomed culture dishes containing minimum essential medium with Earle’s salts supplemented with 10% heat-inactivated fetal bovine serum, 2 mM L-glutamine, 1 mM pyruvate, 20 mM KCl, 10 mM sodium bicarbonate, and 1 mM Hepes (pH 7.2). Following cell attachment (3–6 h after plating), the culture medium was replaced with a neurobasal medium containing B27 supplements (Life Technologies). The cells were cultured in the neuronal cell culture medium for 3 days, and then further cultured in a neuronal cell culture medium (NCM) with or without 20% astrocyte culture media (ACM). Experiments were performed with 4 to 6-day-old cultures; more than 90% of the cells in these cultures were neurons, and the remainder were astrocytes, as judged by the cell morphology and by immunostaining with antibodies against neurofilaments and glial fibrillary acidic protein. Microglial BV-2 cell cultures were prepared as previously described [52]. The BV-2 cells were incubated in the culture medium in a humidified incubator at 37 °C and 5% CO2.

### Sample preparation for two-dimensional electrophoresis (2-DE)

Brain samples were homogenized in liquid nitrogen, after which the sonicated tissues were lysed in buffer (7 M urea, 2 M thiourea, 4% w/v CHAPS, 2.5% DTT buffer, and protease inhibitor [Roche, Cat. no. 11697498001]). The sample mixtures were subsequently centrifuged at 45,000×g at 4 °C for 1 h, after which the protein concentrations were determined by the Bradford protein assay (Bio-Rad, Hercules, CA, USA). In this process, a brain sample was generated from a pool of 6 animals in each group. The pooled samples were analyzed three times.

### 2-DE analysis

One-dimensional isoelectric focusing (IEF) was performed using a 24-cm-immobilized pH gradient (IPG) strips (GE Healthcare, Uppsala, Sweden) in a pH range of 4.0–7.0 (non-linear). Protein (120 μg) was loaded in a total volume of 450 μL. After rehydration for 13 h, the strips were focused at 30 V for 2 h, 100 V for 2 h, 200 V for 1 h, 500 V for 1 h, 1000 V for 1 h, and finally at 8000 V for 22 h to obtain ~ 100,000 VHr (IPGphor; GE Healthcare). Once IEF was completed, the strips were equilibrated in 6 M urea containing 20% glycerol, 2% sodium dodecyl sulfate (SDS), and 0.01% bromophenol blue, with 10 mM tributyl phosphine. Two-dimensional SDS-PAGE was performed using 14% linear gradient acrylamide gels in an Ettan DALT system (GE Healthcare). Proteins were visualized by staining with Coomassie brilliant blue G-250 (Bio-Rad). Only the filtered spots that exceeded an intensity threshold involving a 2-fold increase or decrease among the 3 groups were studied further, whereas the threshold regulation factor for the significance level was set at *P* ≤ 0.05. Finally, the spots showing significant changes in expression were subsequently identified by mass spectrometry.

### Identification of protein spots

The stained gels were scanned with a GS800 densitometer (Bio-Rad) and analyzed using Image master™ (Swiss Institute of Bioinformatics, Geneva, Switzerland). The spots were digested using trypsin, after which the supernatant peptide mixtures were loaded onto a Poros R2 column (Applied Biosystems, Foster City, CA, USA) that had been washed with the following solutions: (i) 70% acetonitrile in 5% formic acid, (ii) 100% acetonitrile, and (iii) 5% formic acid. Peptides were eluted using 5 μL of α-cyano-4-hydroxycinnamic acid and were analyzed with a matrix-assisted laser desorption/ionization time-of-flight (MALDI-TOF) mass spectrometer (Voyager DE-PRO; Applied Biosystems). For protein identification, the masses of the peptides determined by MALDI-TOF were matched with theoretical peptides in the NCBI (http://www.ncbi.nih.gov/) database using the MASCOT (http://www.matrixscience.com) and ProFound programs (http://prowl.rockefeller.edu).

### Western blot analysis

Cells and each area of the brain tissue were homogenized and lysed following 30 min incubation on ice. The lysates were centrifuged at 15,000 rpm for 15 min. An equal amount of total protein (20 mg) isolated from brain tissues was resolved on 10% or 12% SDS-PAGE gels and then was transferred to a nitrocellulose membrane (Hybond ECL; Amersham Pharmacia Biotech, Piscataway, NJ, USA). Blots were blocked for 1 h at room temperature with 5% (w/v) nonfat dried milk in Tris-buffered saline with Tween-20 (TBST) containing 10 mM Tris (pH 8.0), 150 mM NaCl, and 0.05% Tween-20. After a short wash in TBST, the membranes were immunoblotted with the following antibodies: rabbit polyclonal anti-tyrosine hydroxylase (TH), anti-caspase-3 (1:1000 dilution; Cell Signaling Technology, Inc. Beverly, MA, USA), anti-COX-2 (1:1000 dilution; Novus Biologicals, Littleton, CO, USA), anti-PMFBP1 (1:1000 dilution; MyBioSource, San Diego, CA, USA), anti-HMGCL (1:1000 dilution; Abcam, Cambridge, UK), goat polyclonal anti-IBA1 (1:1000 dilution; Abcam, Cambridge, UK), mouse monoclonal anti-BAX, anti-GFAP (1:500 dilution; Santa Cruz Biotechnology Inc., Dallas, TX, USA), or mouse monoclonal anti-iNOS (1:1000 dilution; Cell Signaling Technology, Inc. Beverly, MA, USA). The blots were then incubated with the corresponding horseradish peroxidase-conjugated anti-rabbit (1:5000 dilution; Santa Cruz Biotechnology Inc.), anti-goat (1:10000 dilution; Santa Cruz Biotechnology Inc.), and anti-mouse IgG (1:2000 dilution; Santa Cruz Biotechnology Inc.). Immunoreactive proteins were detected by enhanced chemiluminescence and subjected to densitometric analysis using MyImage (SLB, Seoul, Republic of Korea), and quantified in Labworks 4.0 software (UVP Inc., Upland, CA, USA) [53].

### Immunohistochemistry

While under general anesthesia, the rats received intracardiac perfusion with 50 mL of saline. The brains were fixed in formalin and paraffin-embedded for examination. Tissue sections, 5 μm thick, were used for immunohistochemical examinations. Paraffin-embedded sections were deparaffinized and rehydrated, washed in distilled water, and then subjected to heat-mediated antigen retrieval treatment. Endogenous peroxidase activity was quenched via incubation in 1% hydrogen peroxide in methanol for 30 min, followed by clearing with PBS for 5 min. The sections were blocked for 30 min with 3% normal horse/goat serum diluted in PBS. The sections were then blotted and incubated with primary rabbit anti-TH (1:200 dilution; Cell Signaling Technology, Inc. Beverly, MA, USA), goat anti-IBA1 (1:200 dilution; Abcam, Cambridge, UK) and mouse anti-GFAP (1:200 dilution; Santa Cruz Biotechnology Inc., Dallas, TX, USA) in blocking serum overnight at 4 °C. The next day, the slides were washed three times for 5 min each in PBS and incubated in biotinylated anti-rabbit, anti-goat, or anti-mouse antibodies for 2 h, and then washed again in PBS. The avidin-biotin-peroxidase complexes were formed (Vector Laboratories, Inc., Burlingame, CA, USA) and the peroxidase reaction was developed with diaminobenzidine and peroxide. The tissue sections were then counterstained with hematoxylin, mounted with aqua-mount, and evaluated using a light microscope at × 200 magnification (Olympus, Tokyo, Japan) [54].

### Analysis of TH-positive neurons and fibers

The total number of TH-positive cells was counted in sections using the optical fractionator method for unbiased cell counting as described previously with slight modifications [55]. Briefly, every sixth section throughout the entire extent of the substantia nigra was picked, and immunostaining for TH was performed. The number of TH-positive neurons was counted by using a computer-assisted image analysis system consisting of a Zeiss Axioskop2 Plus photomicroscope equipped with an MS-2000 (Applied Scientific Instrumentation, Eugene, OR, USA) computer-controlled motorized stage, a Sony DXC-390 video camera, a DELL GX260 workstation, and the Optical Fractionator Project module of the BIOQUANT Stereology Toolkit Plug-in for BIOQUANT Nova Prime software (BIOQUANT Image Analysis Corporation, Nashville, TN, USA). The substantia nigra region was observed at low magnification (× 10 objective) and was outlined by using a set of anatomical landmarks. The cell number was counted at high magnification (× 40 objective). The total number of neurons was automatically calculated by the software. For determining striatal TH-positive fiber density, we picked six striatum-containing sections covering the entire head and tail of the striatum from each animal. To prevent non-specific staining, a blocking step was included. Sections were incubated for 2 h at room temperature with 5% BSA in PBS. Sections were then incubated overnight at 4 °C with the primary antibody in blocking solution (5% BSA). TH-positive fiber density was measured by using Bioquant Image Analysis software. All images were converted to a grayscale for standardizing white balance. Each value was corrected for non-specific background by subtracting the optical density of the corpus callosum.

### Measurement of cytokine levels

The lysates of brain tissue were obtained using a protein extraction buffer containing protease inhibitor. IL-1β and IL-6 levels were determined using a specific enzyme-linked immunosorbent assay (ELISA) Kit (ImmunoBiological Laboratories Co., Ltd., Takasaki-Shi, Gunma, Japan). In brief, 100 mL of the sample was added to the precoated plate and was incubated overnight at 4 °C. After washing each well of the precoated plate with washing buffer, 100 mL of labeled antibody solution was added, and the mixture was incubated for 1 h at 4 °C in the dark. After washing, a chromogen was added, and the mixture was incubated for 30 min at room temperature in the dark. Finally, the resulting color was assayed at 450 nm using a microplate absorbance reader (SunriseTM, TECAN, Switzerland) after adding the stop solution.

### Measurement of polyamine content

The polyamine levels in the rat brain were determined using high-performance liquid chromatography. In brief, tissues were sonicated in chilled 0.1 M perchloric acid containing dihydroxybenzylamine as an internal standard. After centrifugation (15,000×*g*, 30 min, 4 °C), the supernatant was diluted with the mobile phase (0.2% acetic acid in distilled water), and 10 μL of sample was isocratically eluted through an 80 × 4.6 mm C18 column (Waters Associates, Milford, MA, USA) at a flow rate of 1.5 mL/min. Polyamines, including spermidine and spermine, were detected using a two-channel electrochemical detector (Waters Associates) at a potential of 1.5 mV. The concentrations were normalized by wet tissue weight. The retention times for spermidine and spermine were 9 and 11 min, respectively.

### Cholesterol level measurement

The total cholesterol level was measured using an enzymatic kit obtained from Roche for use with the Cobas Mira Chemstation (Boehringer Mannheim Corp., Germany). This method follows a two-step approach. In the first step, cholesterol is desterified by the action of cholesterol esterase and subsequently exposed to the action of cholesterol oxidase. The second step involves coupling with a chromogen (color-forming compound), which can be measured using a spectrophotometer, wherein the increase in absorbance due to the chromogen at 405 nm was proportional to the cholesterol concentration in the sample. The amount of total cholesterol was expressed in units of milligrams per deciliter.

### TUNEL assay

DNA fragmentation was examined with terminal deoxynucleotidyl transferase-mediated FITC–dUDP nick-end labeling (TUNEL). TUNEL assays were performed using the In Situ Cell Death Detection Kit (Roche Diagnostics GmbH, Mannheim, Germany) according to the manufacturer’s instructions. In brief, after fixation of 25 mm cryosections with 4% paraformaldehyde, and treatment with 0.1% NaBH_4_ and 0.1% Triton X-100, the slides were incubated for at least 1 h with a reaction mixture containing deoxynucleotidyl transferase and FITC–dUDP (Roche, Reinach, Switzerland). For 4′,6′-diamidino-2-phenylindole dihydrochloride (DAPI) staining, the slides were incubated for 15 min at room temperature in the dark with a mounting medium for fluorescence containing DAPI (Vector Laboratories). The tissues were then examined via fluorescence microscopy (Leica Microsystems AG, Wetzlar, Germany), and the nuclei were visualized via DAPI staining [56].

### Statistical analysis

The image density was measured using the ImageJ software (Wayne Rasband, National Institutes of Health, Bethesda, MD, USA). Graph data were analyzed using the GraphPad Prism version 4 program (GraphPad Software, Inc., San Diego, CA, USA). Data are presented as mean ± standard deviation. Statistical significance was assessed using a two-way analysis of variance (ANOVA). A *P* value of < 0.05 was considered statistically significant.

## Results

### MSC reduces behavioral disorders in double-toxin-induced MSA rats

**Fig. 1:**
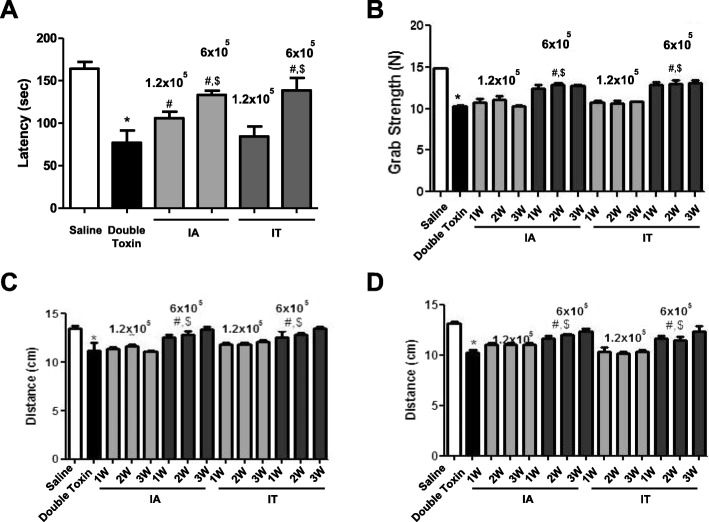
Mesenchymal stem cell (MSC) transplantation ameliorates double-toxin-induced behavior disorders. The performance on the rotarod test was impaired following the double-toxin injection. However, the impairment was ameliorated in the double-toxin-injected MSC-transplanted group (**a**). Double-toxin-induced bradykinesia was ameliorated in the double-toxin-injected MSC-transplanted group (**b**). The stride length of the forelimb (**c**) and hindlimb (**d**) was increased to a greater extent following MSC treatment in the double-toxin injection group. Values are presented as mean ± standard deviation of 10 rats. *P < 0.05, significant difference vs. the saline-injected group. #*P* < 0.05, significant difference among the double-toxin-injected group. $ < 0.05, significant difference between two numbers of the MSC-injected group

To identify whether MSCs can mitigate behavioral disorders caused by the two toxins in MSA, we performed behavioral tests, including rotarod, gait, and grip strength tests. The tests were performed three trials per day for each group (*n* = 10) to obtain statistical significance. The rotarod test was conducted to assess the coordination capability of the rats. Double-toxin injection significantly decreased the latency of falling from the treadmill. However, a significantly better performance in the rotarod test was observed in rats transplanted with MSCs via IA injection (1.2 × 10^5^ cells, 113.5 ± 11.27 s, *F* = 2.57; 6 × 10^5^ cells, 133.6 ± 7.29 s, *F* = 3.45) and IT injections (1.2 × 10^5^ cells, 87.1 ± 19.81 s, *F* = 7.11; 6 × 10^5^ cells, 141.9 ± 21.01 s, *F* = 2.76) (Fig. 1a). Moreover, the grip strength of the forelimb was significantly decreased following double-toxin injection (10.96 ± 0.98 s, *F* = 2.15), although this decrease was mitigated via IA MSC transplantation (1.2 × 10^5^ cells, 11.9 ± 1.34 s, *F* = 5.13; 6 × 10^5^ cells, 13.7 ± 1.18 s, *F* = 1.83) and IT MSC transplantation (1.2 × 10^5^ cells, 11.8 ± 0.91 s, *F* = 2.94; 6 × 10^5^ cells, 13.9 ± 1.01 s, *F* = 4.12, Fig. 1b). In the stride length test, the results showed that the double-toxin injection resulted in shortened forelimb stride length (Fig. 1c) and hindlimb stride length (Fig. 1d). However, the stride length was shortened to a lesser degree in rats with IA MSC transplantation (forelimb 1.2 × 10^5^ cells, 11.7 ± 0.49 s, *F* = 3.62; 6 × 10^5^ cells, 13.8 ± 0.11 s, *F* = 1.15; hindlimb 1.2 × 10^5^ cells, 11.6 ± 0.81 s, *F* = 7.13; 6 × 10^5^ cells, 12.7 ± 1.14 s, *F* = 5.11) and IT MSC transplantation (forelimb 1.2 × 10^5^ cells, 11.9 ± 0.74 s, *F* = 1.11; 6 × 10^5^ cells, 14.1 ± 0.31 s, *F* = 6.09; hindlimb 1.2 × 10^5^ cells, 11.1 ± 0.37 s, *F* = 2.72; 6 × 10^5^ cells, 13.8 ± 0.99 s, *F* = 4.31), as compared to those in double-toxin-injected rats (forelimb 11.04 ± 0.83 s, *F* = 2.66, hindlimb 10.2 ± 0.72 s, *F* = 2.73).

### MSC ameliorates dopaminergic neurodegeneration in double-toxin-induced MSA rats

**Fig. 2:**
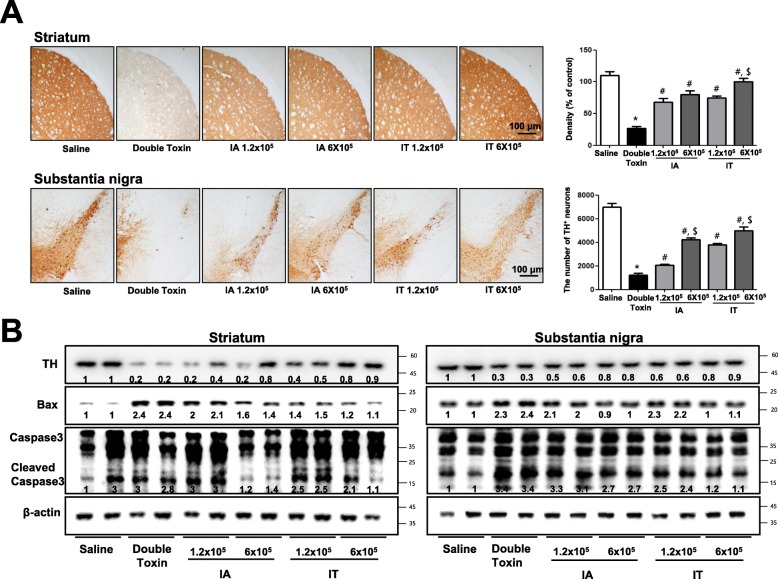
Mesenchymal stem cell (MSC) transplantation ameliorates double-toxin-induced dopaminergic neurodegeneration. The effect of MSC transplantation on TH-positive neurons was measured by western blot and immunohistochemical analysis. The sections of rat striatum and substantia nigra (**a**) incubated with the anti-TH+ primary antibody and biotinylated secondary antibody (*n* = 3). The represented stained tissues were viewed through a microscope (× 50). The graph represents the density of tissue sections and the number of TH-positive neuronal cells. The total number of cells was calculated in each section. The expression of TH and apoptotic proteins (BAX, caspase 3) was examined by using specific antibodies in the brain striatum and substantia nigra (**b**). *P < 0.05, significant difference vs. the saline-injected group (*n* = 10). #*P* < 0.05, significant difference among the double-toxin-injected group (*n* = 10). $ < 0.05, significant difference between two numbers of the MSC-injected group (*n* = 10)

To investigate whether the MSC-induced inactivation of astrocytes could prevent dopamine depletion and neuronal cell death, we evaluated the neurotoxicity of double-toxins using western blots and immunohistochemical staining of TH. Immunohistochemical staining for TH revealed an abundance of TH-positive fibers in the striatum and substantia nigra in the saline-injected group. When the rats were injected with double-toxins, the number of TH-positive neurons was significantly reduced in the substantia nigra. Consistent with this, the density of TH-positive fibers after the double-toxin intoxication was significantly lowered in the striatum (Fig. 2a). However, the population of dopaminergic neurons after double-toxin intoxication was restored in MSC-transplanted rats (Fig. 2a). Consistent with these findings, the double-toxin-induced protein expression of TH in the striatum and substantia nigra was also significantly increased in MSC-transplanted rats, as compared to double-toxin-injected rats (Fig. 2b). We explored whether MSCs could improve dopaminergic neurodegeneration by preventing double-toxin-induced dopaminergic neuronal cell death. In the striatum and substantia nigra regions of the brain, double-toxin injection significantly increased apoptotic cell death, as compared to that noted in control rats (striatum, 81.23 ± 8.75%, *F* = 1.24; nigra, 71.21 ± 7.63%, *F* = 4.11). MSCs inhibited apoptotic cell death in double-toxin-injected rats when injected via IA (striatum 1.2 × 10^5^ cells, 72.16 ± 10.21, *F* = 7.21; 6 × 10^5^ cells, 63.2 ± 7.63 s, *F* = 10.04; nigra 1.2 × 10^5^ cells, 66.12 ± 8.74, *F* = 9.67; 6 × 10^5^ cells, 53.27 ± 8.14, *F* = 4.12) and IT routes (striatum 1.2 × 10^5^ cells, 71.12 ± 3.97, *F* = 3.33; 6 × 10^5^ cells, 37.2 ± 4.48 s, *F* = 4.17, *P* < 0.05; nigra 1.2 × 10^5^ cells, 51.74 ± 9.35, *F* = 11.21; 6 × 10^5^ cells, 29.64 ± 6.28, *F* = 4.32) (Supplementary Fig. 1). To determine whether double-toxin injections can induce neuronal cell death, western blotting was used to detect the expression of apoptotic markers (caspase 3 and BAX) in the rat brains. Our data indicated that the double-toxin-induced cleavage of caspase 3, and the expression of BAX in the striatum and substantia nigra were significantly decreased in MSC-transplanted rats (Fig. 2b).

### MSC exhibits anti-inflammatory effects in double-toxin-induced MSA rats

**Fig. 3:**
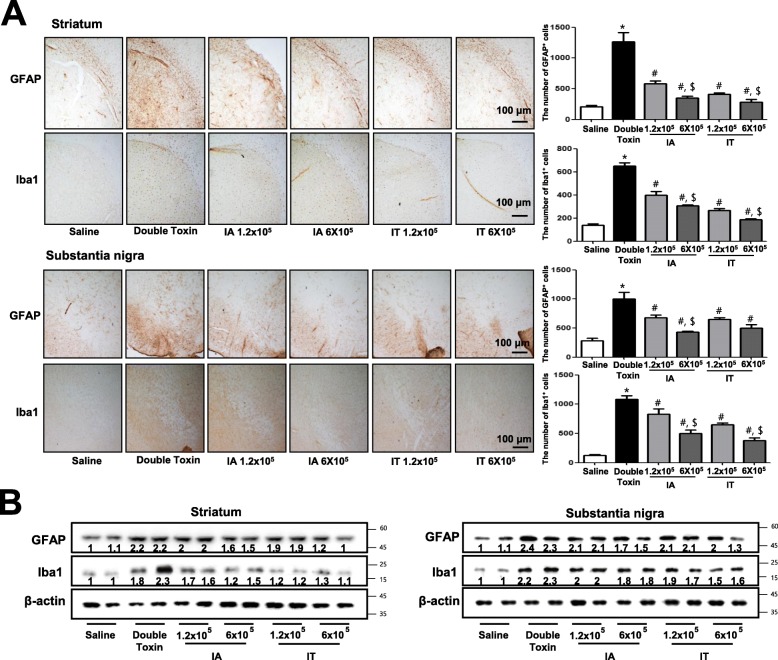
Mesenchymal stem cell (MSC) transplantation reduces the expression of GFAP and IBA1. The effect of MSC transplantation on reactive astrocytes was measured via immunohistochemical analysis and western blotting. The sections of the rat brain (striatum and substantia nigra) incubated with anti-GFAP and anti-IBA1 primary antibody and the biotinylated secondary antibody (*n* = 3). The represented stained tissues were viewed through a microscope (× 50) (**a**). The graph represents the number of GFAP and IBA1-positive cells. The total number of cells was calculated in each section. The expression of GFAP and IBA1 was examined using specific antibodies in the brain (**b**). The values are presented as mean ± standard deviation of 10 rats. *,*P* < 0.05, significant difference vs. the saline-injected group. #*P* < 0.05, significant difference among the double-toxin-injected group. $ < 0.05, significant difference between two numbers of the MSC-injected group

Neuroinflammation is critical to the development of MSA and can be induced through the activation of astrocytes and microglia. To determine whether double-toxin injection can induce neuroinflammation and the activation of astrocytes and microglia, western blotting and immunohistochemistry examinations were used to detect the expression of GFAP (a marker of astrocyte activation) and IBA1 (a marker of microglia activation) in rat brains. Our data showed that the number of reactive cells following immunostaining for GFAP and IBA1 in the striatum and substantia nigra of double-toxin-injected MSC-transplanted rats were significantly lower as compared to those in double-toxin-injected rats (Fig. 3a). The double-toxin-induced protein expressions of GFAP and IBA1 in the striatum and substantia nigra were also significantly decreased in MSC-transplanted rats (Fig. 3b). Moreover, the double toxin-induced protein expressions of inflammatory marker proteins iNOS and COX2 in the striatum and substantia nigra were also significantly decreased in MSC-transplanted rats (Supplementary Fig. 2A). Consistent with these findings, the levels of pro-inflammatory cytokines, IL-1β and IL-6, were significantly decreased in the MSC-transplanted rat striatum and substantia nigra (Supplementary Fig. 2B). In addition, neutrophils were significantly decreased, but WBCs were significantly increased in double-toxin-induced rat blood, but these changes were recovered in MSC-transplanted rat blood (Supplementary Fig. 3A).

### Proteomic analysis of total proteins in the brain striatum

**Fig. 4:**
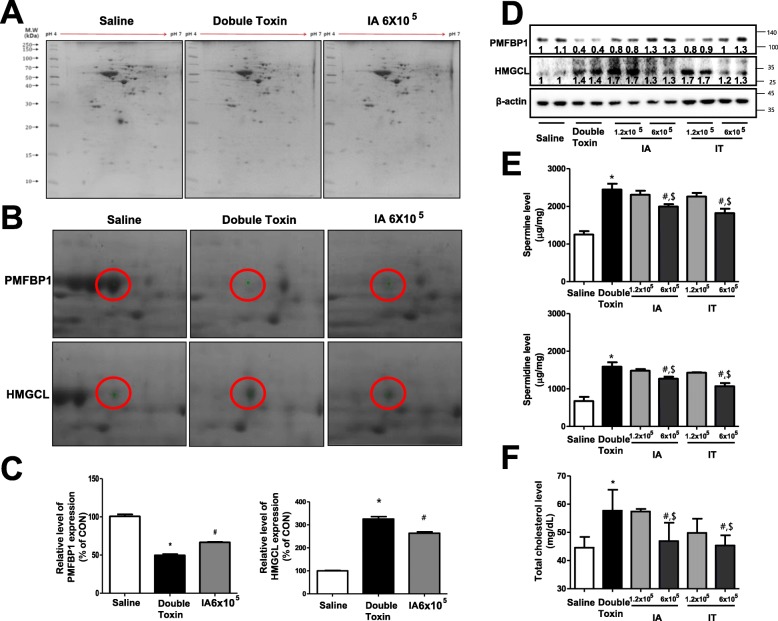
Mesenchymal stem cell (MSC) transplantation restores PMFBP1 and HMGCL expression as well as polyamine content and cholesterol levels in double-toxin-induced MSA rats. Two-dimensional electrophoresis (2-DE) protein patterns in the brain tissues of 3 experimental groups. The brain striatum proteins were examined using 2-DE (**a**). Gel enlargement images (**b**) and relative expression levels of PMFBP1 and HMGCL (**c**) showing differential upregulation between the striatum extracts from 3 experimental group. The expressions of PMFBP1 and HMGCL were examined using specific antibodies in the brain striatum and substantia nigra (**d**). The polyamine levels were measured via high-pressure liquid chromatography (**e**), and the cholesterol levels were measured using CBC tests (**f**). *P < 0.05, significant difference vs. the saline-injected group. #*P* < 0.05, significant difference among the double-toxin-injected group. $ < 0.05, significant difference between two numbers of the MSC-injected group

To characterize the changes in global protein expression in the brain striatum of three experimental groups (sham control, double-toxin-induced, and stem cell-transplanted; IT, 6 × 10^5^ cells), whole proteins extracted from the striatum of three experimental groups were analyzed using 2-DE gels. Computer analysis of the gel images showed good matching in three analytical replicates, including sham-controlled rats, double-toxin-induced rats, and MSC-transplanted rats (IT, 6 × 10^5^). The 2-DE protein maps of the samples from the three groups are shown in Fig. 4a. Approximately, 350 spots were detected in 1 gel from the brain striatum. The spots that showed significantly different expression were selected for further analysis. In total, six spots were identified as representing the differential expression of key proteins in three experimental groups. Furthermore, the six spots were classified into two groups, including the increased-decreased group (upregulation due to double-toxin-induction and downregulation due to MSC transplantation; *n* = 5) and decreased-increased group (downregulation due to double-toxin-induction and upregulation due to MSC transplantation; *n* = 1) based on the expression level. As shown in Table 1, the five increased-decreased proteins included serum albumin precursor, 3-hydroxymethyl-3-methylglutaryl-CoA lyase, the membrane-binding domain of Ctp phosphocholine cytidylyltransferase, GFAP, and alpha tubulin, whereas the decreased-increased protein was polyamine-modulated factor 1-binding protein (PMFBP1) (Table 1).

**Table. 1:** Differentially expressed proteins in three experimental groups

NCBI BLAST	Protein name	Mass	Fold change	Fold change
			By double toxin	By MSC
gi|158138568	Serum albumin precursor [*Rattus norvegicus*]	68,758	2.247	0.517
gi|451770389	3-hydroxymethyl-3-methylglutaryl-CoA cytoplasmic [Rattus norvegicus]	36,556	2.529	0.774
gi|253723061	Nmr structure of the membrane-binding domain of Ctp phosphocholine cytidylyltransferase	2872	2.115	0.948
gi|430721	Glial fibrillary acidic protein [Rattus norvegicus]	49,958	4.068	0.647
gi|223556	tubulin alpha	50,241	3.257	0.259
gi|19705509	Polyamine-modulated factor 1-binding protein 1 [Rattus norvegicus]	113,588	0.547	1.235

### MSCs restored PMFBP1 levels, but reduced HMGCL levels in double-toxin-induced MSA rats

Of these proteins, we were particularly interested in the role of PMFBP1, as it controls the synthesis of polyamines, which are critical to neuronal cell death. Moreover, we were interested in the role of HMGCL, an enzyme involved in the synthesis of cholesterol, which is critical in neurodegenerative disease. To determine the effect of MSC transplantation on the regulation of PMFBP1 and HMGCL expression, we assessed the expressions of PMFBP1 and HMGCL after MSC transplantation. Following MSC transplantation, the PMFBP1 spots demonstrated an increased ratio (Fig. 4b). The volume ratio of this spot was significantly lower in the double-toxin-induced rats (49.69%), as compared to the sham-controlled rats (100.67%). Following MSC transplantation, this volume was markedly increased in the rats transplanted with MSCs (6 × 10^5^ cells) via IT injection (66.58%) (Fig. 4c). However, the volume ratio of HMGCL spots decreased after MSC transplantation (Fig. 4c). In fact, the volume ratio of this spot was significantly higher in double-toxin-induced rats (49.69%) than in the sham-controlled rats (100.67%). Following MSC transplantation, this volume was markedly lower in the rats transplanted with MSCs (6 × 10^5^ cells) via IT injection (66.58%) (Fig. 4c). To determine the expression of PMFBP1 and HMGCL in all the experimental groups, we evaluated the expression of PMFBP1 and HMGCL using western blot analysis. The expression of PMFBP1 in the striatum and substantia nigra was significantly decreased, although the expression of HMGCL was increased in double-toxin-injected rats. However, the expression of PMFBP1 and HMGCL was significantly recovered in MSC-transplanted rats as compared to that in the double-toxin-injected rats (Fig. 4d).

### MSC reduces polyamine content and cholesterol levels in double-toxin-induced MSA rats

PMFBP1 regulates polyamine clearance, which is critical for neuronal cell death. Hence, we measured the polyamine content in the brain using the HPLC system. The content of spermidine and spermine was significantly increased in the double-toxin-induced rats, as compared to that in the sham-controlled rats. Following MSC transplantation, these contents were markedly decreased in the MSC-transplanted rats (Fig. 4e). HMGCL can induce cholesterol synthesis, which is critical in neurodegenerative disease; hence, we measured the cholesterol level in the brain. The total cholesterol level was significantly increased in the double-toxin-induced rats (57.63 ± 7.48 mg/dL) as compared to the sham-controlled rats (44.57 ± 3.84 mg/dL). Following MSC transplantation, this level was markedly decreased in the MSC-transplanted rat brain (IA 1.2 × 10^5^ cells, 57.40 ± 0.9 mg/dL; 6 × 10^5^ cells, 46.90 ± 6.48 mg/dL; IT 1.2 × 10^5^ cells, 49.80 ± 5.03 mg/dL; 6 × 10^5^ cells, 45.33 ± 3.62 mg/dL) (Fig. 4f).

### Knockdown of PMFBP1 and recombinant HMGCL protein reverses the protective effect of co-culture with MSCs in 6-OHDA-induced striatal neuronal cells

**Fig. 5:**
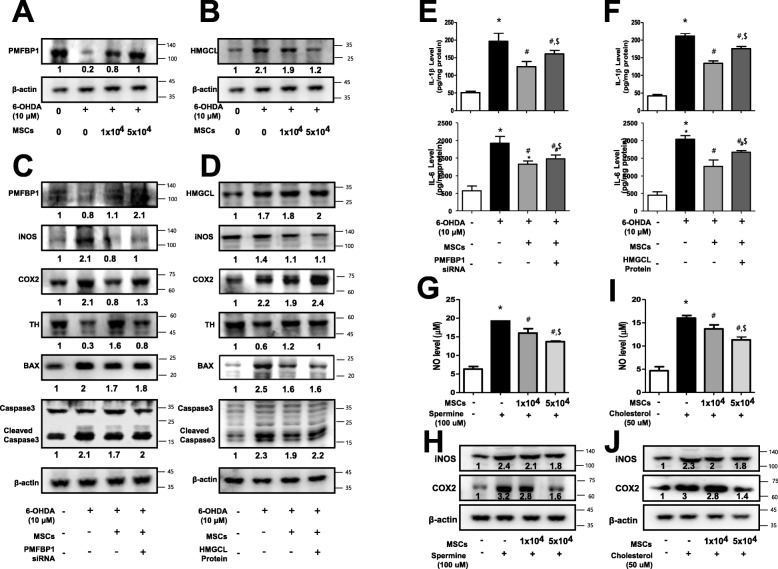
The effect of polyamine and cholesterol on dopaminergic neurodegeneration and apoptosis in mesenchymal stem cells (MSCs) co-cultured with neuronal cells. The promoting effect of MSCs in terms of PMFBP1 (**a**) and HMGCL (**b**) expression were examined using specific antibodies in primary cultured neuronal cells. To assess the involvement of PMFBP1 and the inhibitory effect of MSCs on double-toxin-induced neuro-degeneration, the expression of PMFBP1 was knocked-down in primary cultured neuronal cells using a specific siRNA of PMFBP1 (10 nM) for 24 h at 37 °C. These effects were measured by western blot analysis. The expressions of inflammation marker proteins COX2 and iNOS, apoptotic marker proteins BAX and caspase 3, and TH were examined using specific antibodies (**c**). To assess the involvement of HMGCL on the inhibitory effect of MSCs on double-toxin-induced neuro-degeneration, primary cultured neuronal cells were treated with recombinant HMGCL protein (10 nM) for 24 h at 37 °C. These effects were measured by western blot analysis. The expressions of inflammation marker proteins COX2 and iNOS, apoptosis marker proteins BAX and caspase 3, and TH were examined using specific antibodies (**d**). The release of inflammatory cytokines in PMFBP-1-knockdown (**e**) and HMGCL protein-treated (**f**) neuronal cells was measured using a specific ELISA kit. To assess the effect of MSCs on polyamine- and cholestrol-induced neuroinflammation, the NO levels (**g**, **i**) and expressions of inflammation marker proteins COX2 and iNOS (**h** and **j**) were measured. *P < 0.05, significant difference vs. the saline-injected group. #*P* < 0.05, significant difference among the double-toxin-injected group. $ < 0.05, significant difference between two numbers of MSC-injected group

We also investigated whether the changes in PMFBP1 and HMGCL expression were associated with neuronal cell death, and the modulation of their expression by MSCs is critical for the protective effects on MSA. To identify the changes in PMFBP1 and HMGCL expression on co-culture with MSCs, we evaluated the expression of PMFBP1 and HMGCL by western blot analysis. Following 6-OHDA treatment, the expression of PMFBP1 in the neuronal cells was significantly decreased (Fig. 5a), whereas the expression of HMGCL was significantly increased. However, PMFBP1 and HMGCL expressions were significantly restored (Fig. 5b) in MSCs co-cultured with neuronal cells. In order to further examine the protective mechanisms of PMFBP1 and HMGCL in MSCs against double-toxin-induced neurodegeneration, we knocked-down PMFBP1 expression via siRNA transfection in MSCs co-cultured with neuronal cells and assessed the involvement of PMFBP1 in the inflammatory and apoptotic pathways. Since the expression of PMFBP1 was found to be significantly decreased in PMFBP1-knockdown neuronal cells (Fig. 5c), it was likely that MSCs mediated the suppression of inflammatory protein (iNOS and COX2) expression (Fig. 5c). Moreover, the suppressive effect on the expression of cell death-related proteins BAX and cleaved caspase 3 as well as apoptosis were also reversed in PMFBP1-knockdown neuronal cells (Fig. 5c). Thereafter, we assessed the protective mechanisms of MSCs involving HMGCL against double-toxin-induced neurodegeneration. Accordingly, we treated MSCs co-cultured with neuronal cells with the recombinant HMGCL protein and investigated the involvement of HMGCL in the inflammatory and apoptotic pathways. The MSCs mediated the expression suppression of inflammatory proteins iNOS and COX2 (Fig. 5d). Moreover, the suppressive effect on the expression of cell death-related proteins BAX and cleaved caspase 3 as well as apoptosis were also reversed in HMGCL protein-treated neuronal cells (Fig. 5d). Consistent with these protein expression data, the release of pro-inflammatory cytokines IL-1β and IL-6 were reversed in PMFBP1 knockdown (Fig. 5e) and HMGCL protein-treated (Fig. 5f) neuronal cells.

### Co-culture with MSCs yields anti-inflammatory effects in spermine and cholesterol-induced striatal neuronal cells

To further examine the protection mechanisms of MSCs against polyamine-induced neurodegeneration, we investigated the involvement of MSCs in the inflammatory and apoptotic pathways. Spermine-induced NO release (Fig. 5g) and expression of inflammatory proteins iNOS and COX2 (Fig. 5h) were reduced in a dose-dependent manner when co-cultured with MSCs. Moreover, spermine-induced cell death was also reduced by co-culture with MSCs (Supplementary Fig. 4A). Thereafter, we also investigated the protection mechanisms of MSCs against cholesterol-induced neurodegeneration and examined the involvement of MSCs in the inflammatory and apoptotic pathways. The cholesterol-induced NO release (Fig. 5i) and the expression of inflammatory proteins iNOS and COX2 (Fig. 5j) were reduced in a dose-dependent manner following co-culture with MSCs. Moreover, cholesterol-induced cell death was also reduced following co-culture with MSCs (Supplementary Fig. 4B).

## Discussion

In the present study, we found clear evidence of the protective effects of MSCs on double-toxin-induced MSA. We also found that the transplantation of MSCs prevented neuronal cell death and improved behavioral disorders caused by double-toxin-induced MSA by reducing dopaminergic neurodegeneration and neuroinflammation. Several studies have reported that MSCs could be effective for the treatment of neurodegenerative diseases. The transplantation of human MSCs has been shown to reduce Aβ deposition, improve memory function and alleviate AD pathology in an AD mouse model [57–59]. The co-culture of human MSCs with BV-2 in mouse microglia increased the expression of neprilysin, an Aβ degradation enzyme [60]. Several studies in animal models of PD, which were induced by MPTP and 6-OHDA, have indicated that MSCs can protect and regenerate damaged DA neurons [61–64]. Moreover, MSCs grafted into the striatum, intravenously or intranasally, have been shown to exert neuroprotective effects against nigrostriatal degeneration and to improve motor function in 6-OHDA-induced rats [65]. Double-toxin-induced MSA leads to the loss of neuronal cells in the striatonigral regions. Moreover, upon pathological examination, MSA shows symptoms similar to that of parkinsonism. MSC therapy is effective in treating MSA patients. Finally, a clinical trial using autologous MSC transplantation (4 × 10^7^ cells by IA injection) in patients with MSA indicated improvements in the severity of the symptoms, as well as an attenuation of the decline in cerebral glucose metabolism and gray matter density [66]. Furthermore, several studies have reported that MSCs have a potent effect on immunomodulation and neuroprotection in toxin (MPTP+ 3-NP)-induced (1 × 10^6^ cells/tail vein) or transgenic MSA (5 × 10^5^ cells/tail vein) animal models [67, 68]. MSCs are generally transplanted through the tail vein. However, in the present study, a similar effect was observed for the transplantation of a typical dose (6 × 10^5^ cells) and a lower dose (1.2 × 10^5^ cells) via IT and IA routes. Considering the difference in the species, 1.2 × 10^5^ cells is certainly a low number as compared to that used in other studies. Moreover, we confirmed the similar efficacy between IT administration, which results in less side effects, and IA administration. Furthermore, although other studies isolated MSCs from healthy people, in the present study, MSCs were isolated from patients. Thus, our findings reflect the reduced risk and increased efficacy of using MSC therapy in MSA patients.

Neuroinflammation is considered to be a direct and indirect cause of neuronal cell death and neurodegeneration. The findings of an MSA transgenic mouse model overexpressing α-synuclein may be important in understanding the role of glial cells in neuroinflammation [69–72]. Astrogliosis was observed in all of the examined MSA mice [73]. Moreover, the deficiency of toll-like receptor 4 (TLR-4) in MSA transgenic mouse models was found to enhance the loss of nigrostriatal dopaminergic neurons [74]. These data indicated that neuroinflammation and autoimmune function were critical for the development of MSA. MSCs have been investigated as a therapeutic option for MSA, considering their capacity for differentiation as well as their immunomodulatory and anti-inflammatory properties. In the study involving a transgenic mouse model of MSA [68], the intravenous transplantation of MSCs enhanced the neuroprotective effects in the substantia nigra by regulating the pro-inflammatory cytokine levels in the brain. Similarly, in a double-toxin-induced MSA-P (MPTP- and 3-NP-induced MSA parkinsonism) mouse model, treatment with human MSC (hMSC) improved behavioral disorder, increased neuronal survival, and decreased astro- and microgliosis in the striatum and substantia nigra [67]. In the present study, we demonstrated that MSCs reduced neuroinflammation in a double-toxin-induced MSA model. The effect of MSCs can likely be attributed to the inhibition of the expression of the inflammatory marker proteins COX-2 and iNOS as well as the release of the pro-inflammatory cytokines IL-1β and IL6. In addition, the activation of microglias and astrocytes was significantly increased following the double-toxin induction, although such activation was reduced in MSC-transplanted rats. Evidence suggests that brain inflammation may contribute to the pathology of many neurodegenerative diseases, such as AD, PD, multiple sclerosis, and stroke [75–78]. Microglia and astrocytes play a major role in neuroinflammation through the release of pro-inflammatory cytokines and chemokines as well as the release of NO, which can damage neurons. Thus, these data indicate that MSCs improved behavioral disorder by reducing the neuroinflammatory responses leading to diminished neuronal cell death.

Polyamines also play a major role in the control of cerebral innate immune and inflammatory responses. Polyamines may be involved in reparative efforts of nerve tissue [29] or may play a direct role in the neurodegenerative processes [79–83]. In the brains of AD patients, the mean levels of polyamines were markedly increased by 70% as compared to the levels in normal brains. In addition, the polyamine concentrations were significantly increased in patients with PD and MSA as compared to those in the normal group. Although numerous studies have described the role of polyamine in the development of PD and MSA, the metabolic patterns of polyamines and their role in these diseases remain unclear. In the present study, PMFBP1 expression was significantly decreased in double-toxin-induced MSA rats, and this change led to an increase in polyamine concentration. However, the downregulated PMFBP1 expression was restored following MSC transplantation. The direct binding of polyamines to DNA and their ability to modulate DNA-protein interactions appear to be important in the molecular mechanism of polyamine in facilitating cell death [84, 85]. In mammals, there is a direct relationship between cell death and the levels of polyamines [86]. Caspase-dependent and caspase-independent apoptotic cell death is observed after treatment with several polyamine analogs [87]. Several studies have shown that polyamines accumulate in patients with inflammatory disease and that the metabolism of these polyamines by macrophages leads to neuron damage [88–90]. Hence, some symptoms were treated by reducing the polyamine levels [91–93]. The restoration of PMFBP1 levels by MSCs can lead to a reduction in the accumulated brain polyamines and can accordingly protect neuronal cells against polyamine-induced cell death or inflammation. In fact, we found that MSC transplantation reduced double-toxin-induced polyamine levels in the brain. Moreover, the 6-OHDA-induced neuroinflammation and cell death were significantly reduced by co-culture with MSCs. However, the expressions of the neuroinflammation marker proteins COX-2 and iNOS as well as the release of pro-inflammatory cytokines were increased by knockdown of PMFBP1 expression via siRNA transfection. Moreover, the cleavage of caspase 3 and the expression of BAX were significantly increased by the treatment of PMFBP1 siRNA. Moreover, MSCs rescued spermine-induced NO generation and cell death. These data suggest that enhancing the effects of MSC on PMFBP1 play an important role in the anti-inflammation and renewal of neurons.

HMGCL belongs to the HMG-CoA lyase family, whereas acetyl-CoA acts as a precursor in the first stage of cholesterol synthesis. These reactions occur in the cytosol and begin with the formation of 3-hydroxy-3-methylglutaryl-CoA (HMG-CoA) from acetyl-CoA and acetoacetyl-CoA [94]. Mutations in the gene encoding HMGCL can lead to acetyl-CoA deficiency [95]. The role of cholesterol in neurodegenerative pathology has been reported by many studies. Intriguingly, the α-synuclein protein contains two cholesterol-binding domains [32], and cholesterol seems to modulate α-synuclein aggregation. Other studies have reported that high plasma cholesterol levels lead to an increased risk of PD development [96, 97]. In addition, high cholesterol concentration leads to the production of high levels of reactive oxygen species in the brains of patients with PD [98]. Moreover, the derivatives of cholesterol increase α-synuclein levels and reduce dopamine synthesis [99, 100]. Statins (inhibitors of cholesterol synthesis) strongly reduce the aggregation of α-synuclein in cultured neurons, whereas supplementation of the neurons with exogenous cholesterol increases α-synuclein aggregation and reduces neuronal growth [101]. Furthermore, the treatment of a transgenic mouse model of PD with statins reduced α-synuclein aggregation [102]. Moreover, cholesterol can induce neuroinflammation, and the metabolites of cholesterol may further contribute to neuroinflammation in the brain [103]. A recent study showed that cognitive dysfunction was induced by cholesterol-induced neuroinflammation in mice fed on a high cholesterol diet [104]. In the present study, HMGCL expression was significantly increased in double-toxin-induced MSA but was significantly reduced following MSC treatment. This decrease in HMGCL expression can reduce the accumulation of double-toxin-induced cholesterol, and can protect neuronal cells against cholesterol-induced neuroinflammation. In fact, the double toxin-induced increases in cholesterol levels were significantly reduced by MSC transplantation. Moreover, in our in vitro study, co-culture with MSCs reduced the 6-OHDA-induced cell death and neuroinflammation in cultured neuronal cells. However, the protective effect of MSCs against cell death and inflammation was reduced by treatment with recombinant HMGCL protein. In fact, treatment with recombinant HMGCL protein significantly increased not only the expression of neuroinflammatory markers but also the release of pro-inflammatory cytokines. In addition, the cleavage of caspase 3 and expression of BAX were also increased by treatment with recombinant HMGCL protein. Moreover, MSCs rescued cholesterol-induced cell death and reduced NO generation. These data suggest that the reduced expression of HMGCL following MSC transplantation confers a protective effect against neuroinflammation and cell death.

## Conclusions

MSCs have been recently considered to have successful neuroprotective and immunomodulatory effects on neurological disorders (Supplementary Fig. 5). The significant effect of MSCs on increasing PMFBP1 expression and inhibiting HMGCL expression may represent a promising new therapeutic strategy for MSA. However, further investigation might be necessary to understand the exact mechanism in neuron-specific knockdown on in vivo animal and clinical trials in the following studies of MSA patients, although the knockdown experiments were to assess the involvement of PMFBP1 in neuron for the inhibitory effect of MSCs on double-toxin-induced neuro-degeneration. Taken together, our data suggest that human bone marrow-derived MSCs can improve dopaminergic neuro-degeneration in MSA by reducing polyamine- and cholesterol-modulated neuroinflammation.

## Supplementary information

**Additional file 1: **Supplementary figure 1 Mesenchymal stem cell (MSC) transplantation ameliorates double-toxin-induced neural apoptosis. The effect of MSC transplantation on neuronal apoptosis was measured by TUNEL assay. Detection of apoptotic cell death in the brains as shown by TUNEL staining (*n* = 3). Supplementary figure 2 MSC transplantation ameliorates double-toxin-induced neuroinflammation. Effect of MSC transplantation on neuroinflammation by western blot analysis and ELISA. Expression of iNOS and COX2 was also examined by specific antibodies in the brain striatum and substantia nigra (A). The release of inflammatory cytokines IL-1β and IL-6 in the rat brain striatum and substantia nigra was measured by a specific ELISA kit (B). Each value is presented as mean ± SD of 10 rat. *, *p* < 0.05: significant difference from saline-injected groups and #, p < 0.05: significant difference between the double-toxin-injection groups. Supplementary figure 3 MSC transplantation increases neutrophils but reduces WBC . The cell numbers of neutrophils (A) and WBC (B) were measured using CBC tests. *, *P* < 0.05: significant difference vs. the saline-injected group. #, P < 0.05: significant difference among the double-toxin-injected groups. $ < 0.05: significant difference between two numbers of the MSC-injected groups. Supplementary figure 4 MSC co-culture ameliorates polyamine and cholesterol-induced neuronal cell death. To assess the effects of MSCs on polyamines (A) and cholesterols (B)-induced neuronal cell death, cell viability was measured by the MTT assay. *, p < 0.05: significant difference from the non-treated neuronal cells. #, p < 0.05: significant difference after treatment with spermine (100 μM) or cholesterol (50 μM) in neuronal cells. $, p < 0.05: significant difference between the neuronal cells co-treated with MSCs. Supplementary figure 5 Flowchart for the inhibitory effect of MSCs on double-toxin-induced dopaminergic neurodegenerative MSA.

## Data Availability

Not applicable.

## Abbreviations

6-OHDA: 6-Hydroxydopamine hydrochloride
AD: Alzheimer’s disease
DAPI: Dihydrochloride
GFAP: Glial fibrillary acidic protein expression
HMGCL: 3-Hydroxymethyl-3-methylglutaryl-CoA lyase
LPS: Lipopolysaccharide
MSA: Multiple system atrophy
MSCs: Mesenchymal stem cells
PD: Parkinson’s disease
PMFBP1: Polyamine modulating factor-binding protein 1
SSAT1: Spermidine/spermine N1-acetyltransferase
TUNEL: Terminal deoxynucleotidyl transferase-mediated FITC–dUDP nick-end labeling
