# Editorial Expression of Concern: Prevention of multiple system atrophy using human bone marrow-derived mesenchymal stem cells by reducing polyamine and cholesterol-induced neural damages

**DOI:** 10.1186/s13287-025-04787-4

**Published:** 2025-11-06

**Authors:** Kyung-Ran Park, Chul Ju Hwang, Hyung-Mun Yun, In Jun Yeo, Dong-Young Choi, Pil-Hoon Park, Hyung Sook Kim, Jung Tae Lee, Young Suk Jung, Sang-Bae Han, Jin Tae Hong

**Affiliations:** 1 Department of Oral and Maxillofacial Pathology, School of Dentistry, Kyung Hee University, Seoul, 02453 Republic of Korea; 2 College of Pharmacy and Medical Research Center, Chungbuk National University, 194-31, Osongsangmyeong1-ro, Heungdeok-gu, Cheongju, Chungbuk 361-951 Republic of Korea; 3 College of Pharmacy, Yeungnam University, 280, Daehak-ro, Gyeongsan, Gyeongbuk 712-749 Republic of Korea; 4 Corestem Inc, Pangyo-ro 255 beon-gil, Bundang-gu, Seongnam-si, Gyeonggi 13486 Republic of Korea; 5 College of Pharmacy, Pusan National University, Geumjeong-gu, Busan, Republic of Korea

**Editorial Expression of Concern: Stem Cell Research & Therapy (2020) 11:63** 10.1186/s13287-020-01590-1

The Editors-in-Chief would like to inform the readers that following the publication of this article, concerns have been raised regarding the similarity of Fig. 3b Substantia nigra b-actin blot with Fig. 1c of [1]. The authors were unable to provide raw data for further validation.

The readers are therefore alerted to take caution while interpreting this content.

Hyung-Mun Yun and KyungRan Park agree with this Editorial Express of Concern. None of the remaining authors has responded to correspondence from the Publisher about this Editorial Express of Concern.
