# Supplementary material for: Prevention of multiple system atrophy using human bone marrow-derived mesenchymal stem cells by reducing polyamine and cholesterol-induced neural damages
Source: Stem Cell Res Ther. 2020 Mar 4;11:63. doi: 10.1186/s13287-020-01590-1 (PMC7055099; doi:10.1186/s13287-020-01590-1)

## Supplementary figure legend

**Supplementary figure 1. Mesenchymal stem cell (MSC) transplantation ameliorates double-toxin-induced neural apoptosis.** The effect of MSC transplantation on neuronal apoptosis was measured by TUNEL assay. Detection of apoptotic cell death in the brains as shown by TUNEL staining (n=3).

**Supplementary figure 2. MSC transplantation ameliorates double-toxin-induced neuroinflammation.** Effect of MSC transplantation on neuroinflammation by western blot analysis and ELISA. Expression of iNOS and COX2 was also examined by specific antibodies in the brain striatum and substantia nigra (A). The release of inflammatory cytokines IL-1β and IL-6 in the rat brain striatum and substantia nigra was measured by a specific ELISA kit (B). Each value is presented as mean ± SD of 10 rat. *, $p<0.05$: significant difference from saline-injected groups and #, $p<0.05$: significant difference between the double-toxin-injection groups.

**Supplementary figure 3. MSC transplantation increases neutrophils but reduces WBC .** The cell numbers of neutrophils (A) and WBC (B) were measured using CBC tests. *, $P<0.05$: significant difference vs. the saline-injected group. #, $P<0.05$: significant difference among the double-toxin-injected groups. $<0.05: significant difference between two numbers of the MSC-injected groups.

**Supplementary figure 4. MSC co-culture ameliorates polyamine and cholesterol-induced neuronal cell death.** To assess the effects of MSCs on polyamines (A) and cholesterols (B)-induced neuronal cell death, cell viability was measured by the MTT assay. *, $p < 0.05$: significant difference from the non-treated neuronal cells. #, $p < 0.05$: significant difference after treatment with spermine (100 μM) or cholesterol (50 μM) in neuronal cells. $, $p < 0.05$: significant difference between the neuronal cells co-treated with MSCs.

**Supplementary figure 5. Flowchart for the inhibitory effect of MSCs on double-toxin-induced dopaminergic neurodegenerative MSA.**

# Supplementary figure 1.

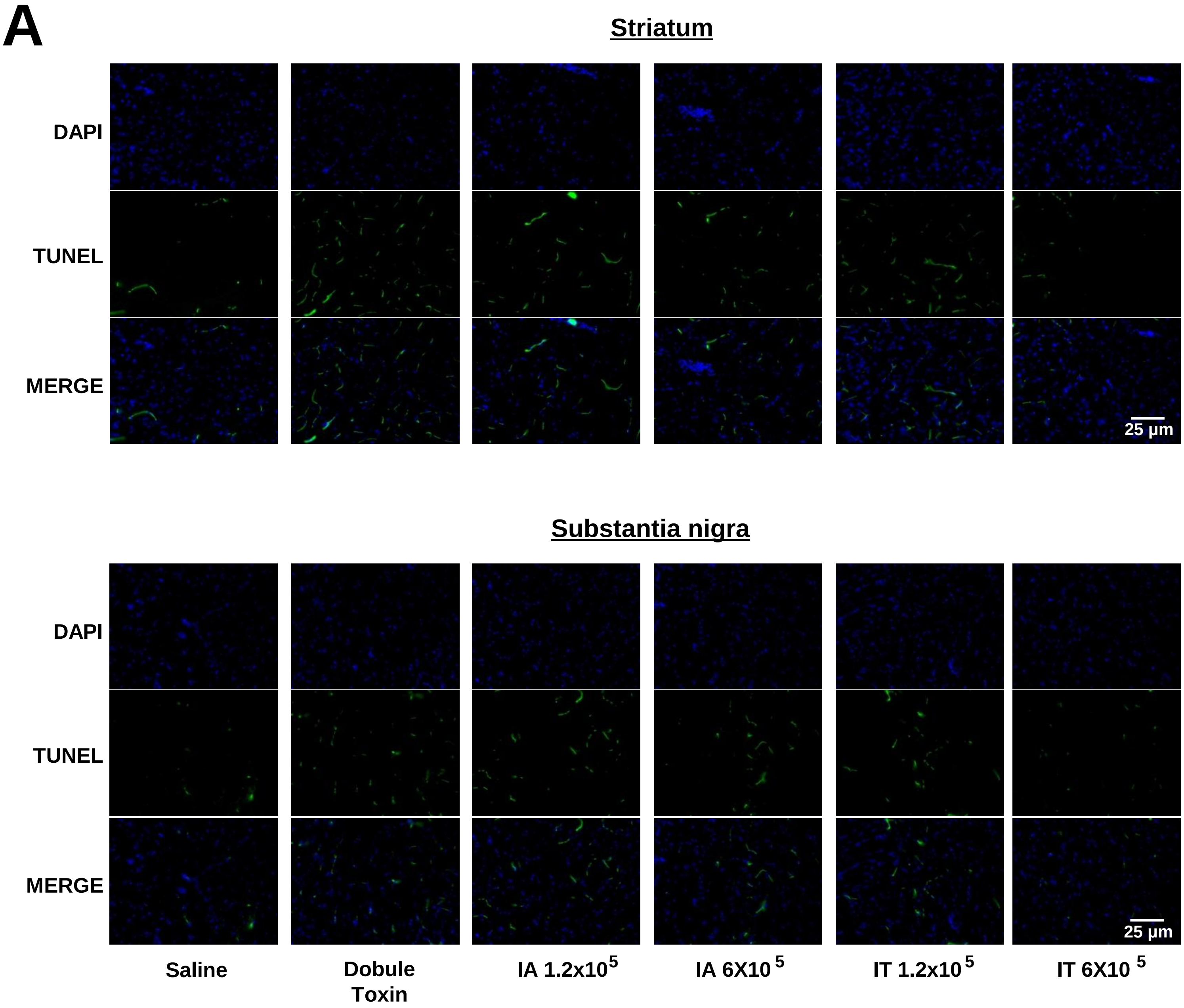

## Supplementary figure 2.

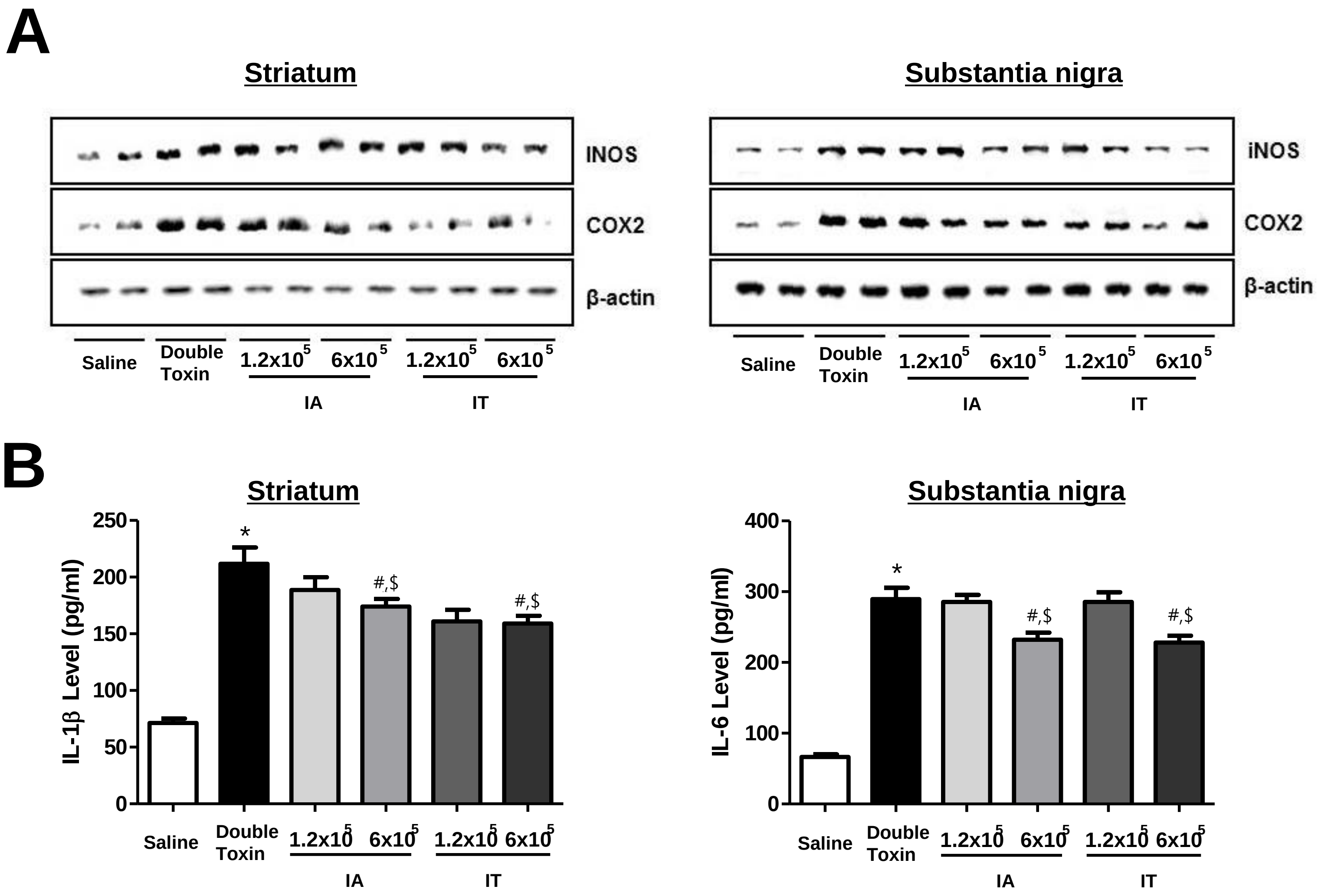

**Supplementary figure 3.**

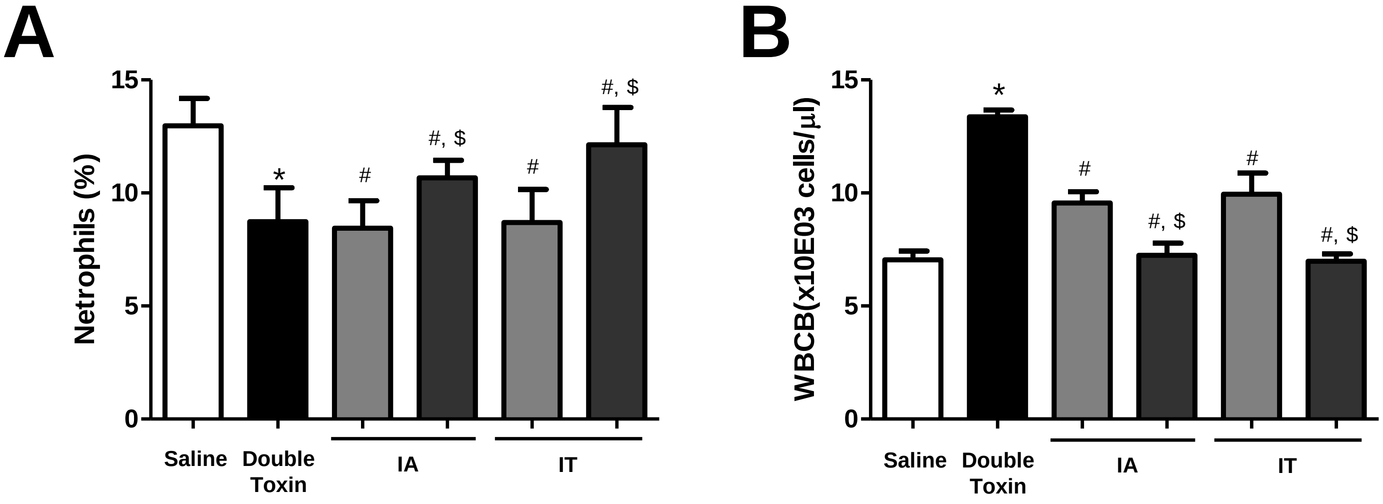

**Supplementary figure 4.**

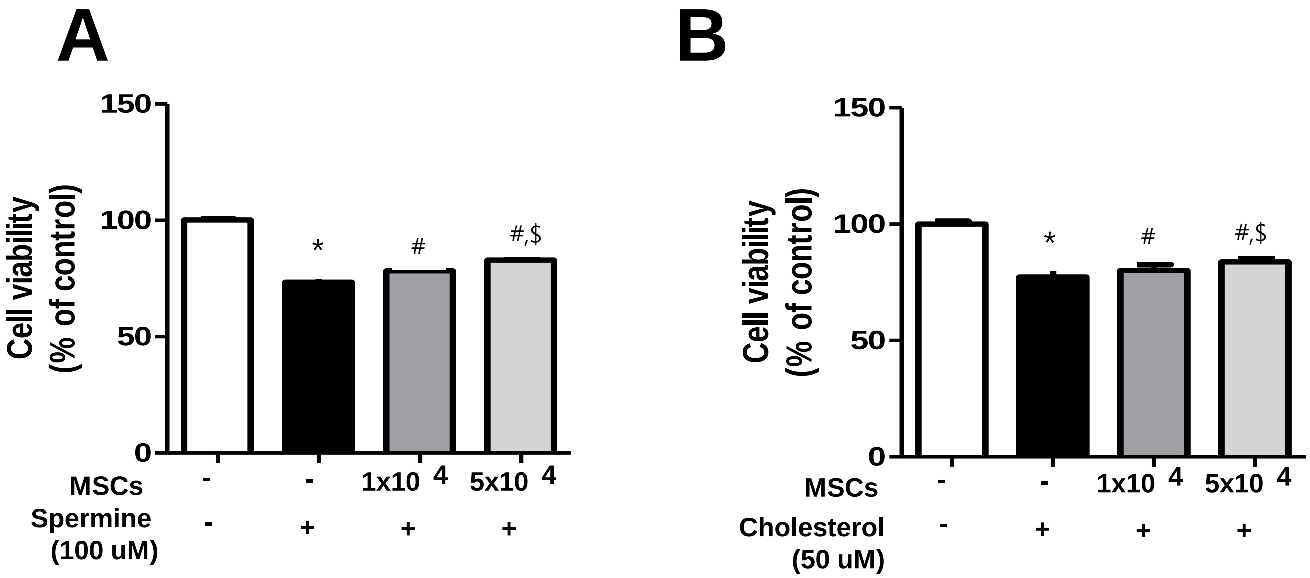

**Supplementary figure 5.**

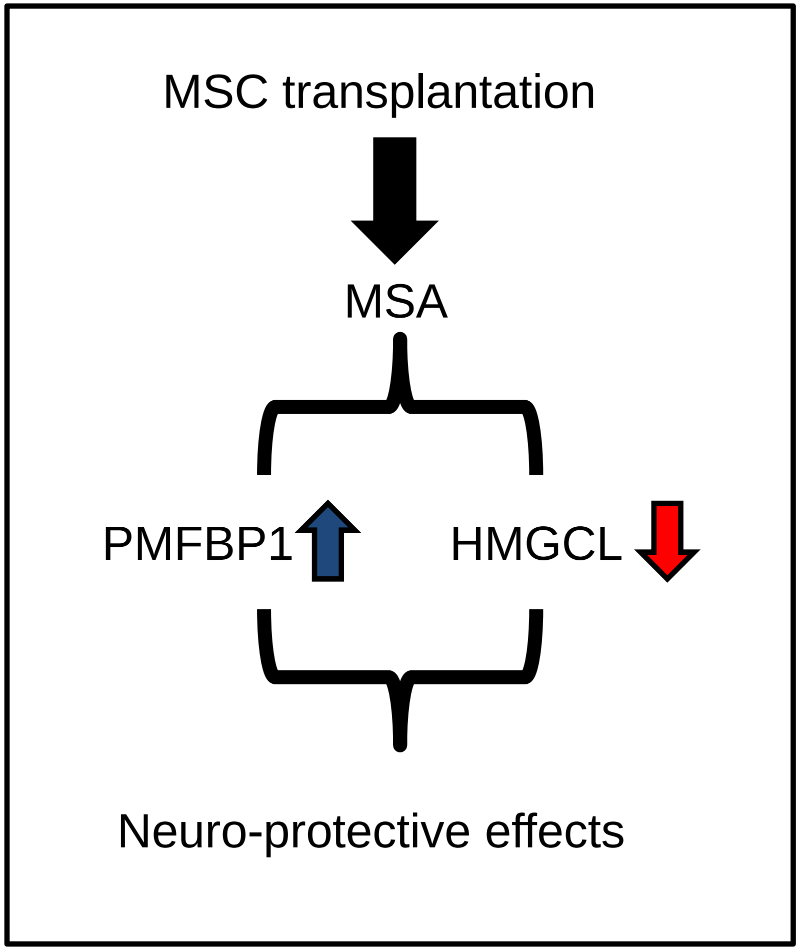

Supplement: Supplementary file 1 — Additional file 1: Supplementary figure 1 Mesenchymal stem cell (MSC) transplantation ameliorates double-toxin-induced neural apoptosis. The effect of MSC transplantation on neuronal apoptosis was measured by TUNEL assay. Detection of apoptotic cell death in the brains as shown by TUNEL staining (n = 3). Supplementary figure 2 MSC transplantation ameliorates double-toxin-induced neuroinflammation. Effect of MSC transplantation on neuroinflammation by western blot analysis and ELISA. Expression of iNOS and COX2 was also examined by specific antibodies in the brain striatum and substantia nigra (A). The release of inflammatory cytokines IL-1β and IL-6 in the rat brain striatum and substantia nigra was measured by a specific ELISA kit (B). Each value is presented as mean ± SD of 10 rat. *, p < 0.05: significant difference from saline-injected groups and #, p < 0.05: significant difference between the double-toxin-injection groups. Supplementary figure 3 MSC transplantation increases neutrophils but reduces WBC . The cell numbers of neutrophils (A) and WBC (B) were measured using CBC tests. *, P < 0.05: significant difference vs. the saline-injected group. #, P < 0.05: significant difference among the double-toxin-injected groups. $ < 0.05: significant difference between two numbers of the MSC-injected groups. Supplementary figure 4 MSC co-culture ameliorates polyamine and cholesterol-induced neuronal cell death. To assess the effects of MSCs on polyamines (A) and cholesterols (B)-induced neuronal cell death, cell viability was measured by the MTT assay. *, p < 0.05: significant difference from the non-treated neuronal cells. #, p < 0.05: significant difference after treatment with spermine (100 μM) or cholesterol (50 μM) in neuronal cells. $, p < 0.05: significant difference between the neuronal cells co-treated with MSCs. Supplementary figure 5 Flowchart for the inhibitory effect of MSCs on double-toxin-induced dopaminergic neurodegenerative MSA. [file 13287_2020_1590_MOESM1_ESM.pdf]
